# Origin-Associated Variation in Volatile Profiles of Commercial Sour Jujube Pulp

**DOI:** 10.3390/foods15183318

**Published:** 2026-09-19

**Authors:** Nannan Chen, Xianglong Wu, Oujun Dai, Beizhi Zhang, Shujing Xuan, Fuzhi Xie, Yu Chen, Fengzhong Wang, Liang Zhang

**Affiliations:** 1 National Technology Innovation Center for Saline-Alkali Agriculture, Dongying 257000, China; chennan0114@gmail.com; 2 Institute of Food Science and Technology, Chinese Academy of Agricultural Sciences, Beijing 100193, China; 13145216783@163.com (X.W.); d15158590879@163.com (O.D.); zhangbeizhi777@163.com (B.Z.); xuanshujing10@163.com (S.X.); xiefuzhi2002@163.com (F.X.); cynthia1070@163.com (Y.C.); 3 College of Food Science and Technology, Huazhong Agricultural University, Wuhan 430070, China; 4 College of Food Science and Technology, Hebei Agricultural University, Baoding 071000, China

**Keywords:** sour jujube, *Ziziphus jujuba* var. *spinosa*, olatile profiles, HS-SPME-GC-MS, odor activity value (OAV), OPLS-DA, reported geographical origin, origin-associated variation

## Abstract

Sour jujube (*Ziziphus jujuba* Mill. var. *spinosa*) is an ecologically and economically important fruit widely utilized in the functional food industry. However, the geographical variation in its volatile flavor profile and the potential odor relevance of individual volatile features remain insufficiently understood. In this study, volatile profiles of commercial sour jujube pulp samples labeled as originating from eight representative geographical regions across China were characterized using headspace solid-phase microextraction coupled with gas chromatography–mass spectrometry (HS-SPME-GC-MS). A total of 1296 putative volatile annotations were obtained, including esters, aldehydes, alcohols, acids, ketones, terpenoids, hydrocarbons, and other compounds. Principal component analysis (PCA) and orthogonal partial least squares-discriminant analysis (OPLS-DA) revealed distinct distribution patterns among several sample groups. The OPLS-DA model showed acceptable internal validation performance, with cumulative R^2^X, R^2^Y, and Q^2^ values of 0.967, 0.891, and 0.811, respectively. Medium-chain fatty acid ethyl esters, including ethyl dodecanoate, ethyl decanoate, and ethyl octanoate, contributed strongly to the multivariate differentiation of SDDY samples. Odor activity value (OAV) analysis further indicated that volatile abundance was not necessarily proportional to predicted odor contribution. Several trace volatile features with low reported odor thresholds, such as 1-nonen-3-one, 2-methoxy-3-(2-methylpropyl)pyrazine, and benzenemethanethiol, showed high calculated OAVs and may be important aroma-relevant candidates. Overall, this study provides an integrated volatile-profiling and OAV-based approach for characterizing compositional differences among commercial sour jujube pulp samples from different reported origins. Because the samples were commercially sourced and no independent external validation or direct sensory evaluation was performed, the results should be interpreted as exploratory evidence of origin-associated differentiation rather than as a validated geographical authentication system.

## 1. Introduction

Sour jujube (*Ziziphus jujuba* Mill. var. *spinosa*) is an ecologically and economically important plant widely distributed in China [1]. Its pulp contains diverse bioactive components, including polysaccharides, organic acids, and phenolic compounds, which are associated with pharmacological properties such as sedative, hypnotic, and antioxidant activities [2,3]. With increasing consumer demand for natural health-promoting products, sour jujube pulp has gained growing attention as a raw material for functional beverages, snacks, and dietary supplements [4].

Among the quality attributes of sour jujube pulp, volatile flavor composition is an important factor affecting sensory quality and consumer acceptance [5,6]. Volatile organic compounds (VOCs), including esters, aldehydes, alcohols, ketones, acids, terpenoids, and other compounds, collectively contribute to fruit aroma characteristics. Headspace solid-phase microextraction coupled with gas chromatography–mass spectrometry (HS-SPME-GC-MS) has been widely used for volatile profiling because it allows efficient enrichment and detection of complex volatile compounds in fruit matrices [7].

Volatile profiles can vary among samples from different geographical or production backgrounds. Such variation may be associated with multiple factors, including genotype, cultivation environment, fruit maturity, harvest time, storage, and postharvest handling [8,9,10]. Previous studies have applied volatile profiling approaches to characterize regional or cultivar-related differences in jujube fruits. For example, HS-SPME-GC/MS and GC-IMS have been used to compare volatile profiles of winter jujube from different regions [11], and GC-IMS combined with multivariate analysis and sensory evaluation has been applied to investigate the volatile profile of red jujube [12]. These studies indicate that volatile profiling is useful for describing compositional variation in jujube-related materials. However, the volatile composition and potential odor relevance of sour jujube pulp from different reported geographical origins remain insufficiently characterized.

In addition, many volatile-profile studies rely mainly on compound abundance, whereas odor perception depends not only on concentration but also on odor threshold and matrix-related effects [13,14]. Odor activity value (OAV) analysis provides a useful theoretical approach for estimating the potential odor contribution of volatile compounds, although it does not replace direct sensory evaluation or GC–olfactometry [15]. Therefore, integrating multivariate volatile profiling with OAV analysis may provide a more comprehensive view of both compositional differentiation and aroma-relevant candidate features.

Accordingly, the present study characterized the volatile profiles of commercial sour jujube pulp samples labeled as originating from eight geographical regions in China, including SDDY, LNCY, HNAY, HBXT, SXLF, HBTHS, SDLW, and XJAKS. HS-SPME-GC-MS was combined with principal component analysis (PCA), orthogonal partial least squares-discriminant analysis (OPLS-DA), variable importance in projection (VIP) analysis, and OAV evaluation. The objectives were to compare volatile-profile patterns among the sample groups, identify candidate discriminatory volatile features, and estimate aroma-relevant features based on calculated OAVs. Because the materials were commercially sourced and external validation was not performed, the results were interpreted as exploratory evidence of origin-associated differentiation rather than as a validated geographical authentication system.

## 2. Materials and Methods

### 2.1. Plant Materials and Sample Sources

Sour jujube (*Ziziphus jujuba* Mill. var. *spinosa*) samples were commercially obtained from suppliers in China. According to the geographical origin information provided by the suppliers, the samples were designated as SDDY (Dongying, Shandong), LNCY (Chaoyang, Liaoning), HNAY (Anyang, Henan), HBXT (Xingtai, Hebei), SXLF (Linfen, Shanxi), HBTHS (Baoding, Hebei), SDLW (Laiwu, Shandong), and XJAKS (Aksu, Xinjiang). The pulp was used for the subsequent volatile profiling analysis.

Because the materials were commercially sourced rather than collected directly from orchards, detailed information concerning individual trees, the number of fruits collected per tree, harvest conditions, and independently quantified maturity indices was not available. For each reported geographical origin, six parallel subsamples were independently prepared and subjected separately to the complete HS-SPME-GC-MS analytical procedure, as described below.The supplier-reported geographical origins and corresponding regional environmental characteristics of the commercial sour jujube samples are summarized in Table 1.

**Table 1:** Supplier-reported geographical origins and corresponding regional environmental characteristics of the commercial sour jujube samples.

Sample Code	Geographical Origin	Longitude (E)	Latitude (N)	Altitude (m)	MAT (°C)	MAP (mm)
SDDY	Dongying, Shandong	118.67°	37.43°	5	13.5	560
LNCY	Chaoyang, Liaoning	120.45°	41.58°	170	8.5	480
HNAY	Anyang, Henan	114.35°	36.10°	70	14	580
HBXT	Xingtai, Hebei	114.50°	37.07°	60	13.5	520
SXLF	Linfen, Shanxi	111.52°	36.08°	450	12	500
HBTHS	Baoding, Hebei	115.47°	38.87°	20	12.5	550
SDLW	Laiwu, Shandong	117.67°	36.20°	200	13	700
XJAKS	Aksu, Xinjiang	80.27°	41.17°	1100	10	75

The geographical origins were obtained from information provided by the suppliers. Longitude, latitude, altitude, mean annual temperature (MAT), and mean annual precipitation (MAP) represent the corresponding reported production regions and were used for descriptive purposes only.

### 2.2. Sample Preparation and Headspace Solid-Phase Microextraction (HS-SPME)

Samples retrieved from −80 °C were directly ground to a homogeneous powder in liquid nitrogen using a laboratory ball mill (MM400, Retsch, GmbH, Haan, Germany). For each reported geographical origin, six parallel subsamples were independently prepared. Approximately 500 mg of each subsample was accurately weighed and transferred into a 20 mL headspace vial. Each subsample was then subjected independently to the complete sample preparation and HS-SPME-GC-MS procedure. Accordingly, the six measurements for each origin were treated as independently prepared analytical replicates (*n* = 6). Subsequently, 2 mL of saturated sodium chloride (NaCl) solution and 20 μL of an isotopically labeled internal standard solution, 3-hexanone-2,2,4,4-d4 (10 μg/mL; CAS No. 24588-54-3), were added before HS-SPME extraction [16]. The vial was then sealed using crimp-top caps with TFE-silicone headspace septa (Agilent Technologies, Santa Clara, CA, USA).

The fully automated HS-SPME extraction process was executed utilizing an SPME Arrow system integrated with an autosampler, a fiber conditioning station, and an agitator (CTC Analytics AG, Zwingen, Switzerland). The sample vial was incubated and agitated at 60 °C for 5 min [17]. Then, a 120 μm DVB/CWR/PDMS SPME Arrow fiber (Agilent) was exposed to the headspace of the vial to absorb volatile organic compounds (VOCs) under continuous thermal conditions at 60 °C for 15 min. The 120 μm DVB/CWR/PDMS SPME Arrow was selected as part of a previously optimized broad-coverage volatile profiling workflow established by the analytical service provider to achieve broad volatile coverage and stable analytical performance. This fiber condition was not specifically optimized for sour jujube pulp in the present study. Prior to sampling, the SPME fiber was automatically conditioned in the conditioning station at 250 °C for 5 min. Finally, the fiber was inserted into the GC injector port at 250 °C for a 5 min thermal desorption. System blank headspace vials were included in the analytical sequence to monitor potential residual contamination and carryover during HS-SPME-GC-MS analysis. Blank peak areas were exported and inspected as part of analytical quality control. Because blank-based background subtraction or feature filtering was not applied in the processed dataset supplied by the analytical service provider, the blank values were not used to retrospectively correct the semi-quantitative concentrations. The blank peak-area information is provided in Table S6 for transparency.

### 2.3. GC-MS Analysis

GC-MS analysis was performed using an Agilent 8890 gas chromatograph coupled to an Agilent 7000D triple-quadrupole mass spectrometer (Agilent Technologies, Santa Clara, CA, USA), operated in selected ion monitoring (SIM) mode. Chromatographic separation was achieved using a DB-5MS capillary column (30 m × 0.25 mm × 0.25 μm; Agilent J&W Scientific, Folsom, CA, USA). High-purity helium (≥99.999%) was used as the carrier gas at a constant flow rate of 1.2 mL/min. The inlet temperature was maintained at 250 °C with a solvent delay of 3.5 min. The oven temperature program was as follows: 40 °C for 3.5 min, increased to 100 °C at 10 °C/min, then to 180 °C at 7 °C/min, and finally to 280 °C at 25 °C/min, followed by a 5 min hold.

The mass spectrometer was operated in electron ionization (EI) mode at 70 eV. The ion-source, quadrupole, and transfer-line temperatures were maintained at 230, 150, and 280 °C, respectively. Data were acquired in SIM mode. For each volatile feature, one quantitative ion and the corresponding qualitative ion(s) were monitored according to the established in-house database.

### 2.4. Volatile Annotation and Semi-Quantification

Volatile features were annotated using an established in-house qualitative database. For qualitative annotation, chromatographic deconvolution and spectral matching were performed using MassHunter software (version B.08.00; Agilent Technologies). The peak width was set to 20; the resolution, sensitivity, and chromatographic peak-shape settings were set to medium; and the minimum spectral match factor was set to 70. Reference mass spectra were compared with the NIST 2020 mass spectral library. Selected entries in the in-house database were additionally supported by authentic standards, and quantitative and qualitative ions were used to support feature annotation during SIM acquisition.

Retention indices were used as an additional criterion for compound annotation. Experimental retention indices (RIs) were determined using a C7–C40 n-alkane mixture analyzed under the same chromatographic conditions and were calculated according to the Van den Dool and Kratz equation [18]:

$$RI=100z+100\left(\frac{t_{R\left(x\right)}-t_{R\left(z\right)}}{t_{R\left(z+1\right)}-t_{R\left(z\right)}}\right)$$

where $t_{R}\left(x\right)$ is the retention time of the analyte, and $t_{R}\left(z\right)$ and $t_{R}\left(z+1\right)$ are the retention times of the n-alkanes containing z and z+1 carbon atoms eluting immediately before and after the analyte, respectively. Agreement within ±30 RI units between the experimental RI and the corresponding reference RI was considered consistent with the proposed annotation. Compound-specific retention times and experimental RI values for 30 representative volatile features are provided in Table S5, whereas diagnostic ions, reference NIST RI values, and spectral match factors for the full volatile-feature dataset are provided in Table S4. Because authentic-standard support was available only for selected entries in the in-house database, features without documented standard support were conservatively treated as putatively annotated volatile features rather than unequivocally identified compounds.

Semi-quantification of individual volatile features was performed using a single internal-standard method [19]. The isotopically labeled compound 3-hexanone-2,2,4,4-d4 (CAS No. 24588-54-3) was used as the internal standard. As a deuterated, non-native reference compound, it was added before HS-SPME extraction and served as a common reference for normalizing variability associated with sample extraction and instrumental response. Semi-quantitative contents were calculated according to the following equation:

$$X_{i}=\left(V_{s}\times \frac{C_{s}}{M}\right)\times \left(\frac{I_{i}}{I_{s}}\right)\times 10^{-3}$$

where $X_{i}$ is the semi-quantitative content of volatile feature i (μg/g); $V_{s}$ is the volume of internal-standard solution added (μL); $C_{s}$ is the concentration of the internal standard (μg/mL); M is the sample mass (g); $I_{i}$ is the peak area of volatile feature i; and $I_{s}$ is the peak area of the internal standard. Because a single internal standard rather than compound-specific calibration curves was used, the resulting values were interpreted as semi-quantitative estimates rather than absolute concentrations.

Pooled quality-control (QC) samples were prepared by combining aliquots of the analytical samples and were inserted at approximately one QC sample per 10 analytical samples to monitor analytical repeatability. Technical stability was evaluated based on the overlap of QC total-ion chromatograms and the coefficient-of-variation (CV) distribution of detected volatile features. Features with a QC coefficient of variation (CV) < 0.5 (50%) were retained in the final processed dataset.

### 2.5. Odor Activity Value (OAV) Calculation

Odor activity values (OAVs) were calculated to estimate the potential odor contribution of individual volatile features [20,21]. For each analytical replicate, OAV was calculated according to the following equation:

$$OAV_{i}=\frac{C_{i}}{T_{i}}$$

where $C_{i}$ represents the semi-quantitative concentration of volatile feature i (μg/g for the solid pulp samples), and $T_{i}$ represents the corresponding reported odor threshold. The semi-quantitative concentrations were obtained using the internal-standard method. Odor descriptions were obtained from Flavornet [22], and the odor-threshold values used for OAV calculation were referenced to van Gemert, *Odour Thresholds: Compilations of Odour Threshold Values in Air, Water and Other Media* [20]. Compound-specific threshold matrices were not provided in the original analytical dataset and therefore were not inferred. Features with OAV ≥ 1 were considered potentially odor-active. Because the concentrations were obtained by semi-quantification and no direct sensory evaluation or GC-O analysis was performed, OAV was interpreted as a theoretical indicator of potential odor contribution rather than a direct measure of sensory perception [15].

### 2.6. Statistical Analysis

Six independently prepared analytical replicates were analyzed for each reported geographical group (n = 6). Principal component analysis (PCA) was performed using OriginPro (OriginLab Corporation, Northampton, MA, USA) based on the replicate-level volatile profiles to visualize the overall distribution of the samples. Orthogonal partial least squares-discriminant analysis (OPLS-DA) was performed using SIMCA 14.1 (Sartorius Stedim Data Analytics AB, Umeå, Sweden) to further evaluate supervised differentiation among the sample groups [23]. The OPLS-DA model comprised seven predictive components and one orthogonal component. Model performance was evaluated using the cumulative goodness-of-fit parameters R^2^X and R^2^Y and the predictive ability parameter Q^2^. Q^2^ was estimated using seven-fold cross-validation implemented in SIMCA. Model stability and the potential for overfitting were further assessed using a 200-iteration permutation test. Variable importance in projection (VIP) scores were extracted from the OPLS-DA model to evaluate the contribution of individual volatile features to group differentiation. Features with VIP > 1 were considered important contributors to the model. Because orchard-level biological replicates were not available, the multivariate results were interpreted as differentiation within the present commercial sample set rather than as definitive evidence of geographical causation [24].

## 3. Results

### 3.1. Overall Profile of Volatile Organic Compounds (VOCs) in Sour Jujube Pulp

HS-SPME-GC-MS profiling revealed a chemically diverse volatile composition in the commercial sour jujube pulp samples representing eight supplier-reported geographical origins. The processed dataset contained 1296 putative volatile annotations, including 252 esters, 94 aldehydes, 141 alcohols, 71 acids, 163 ketones, 180 terpenoids, 73 hydrocarbons, and 322 annotations classified as “others”. The latter category comprised heterocyclic compounds, phenols, amines, aromatics, ethers, nitrogen-containing compounds, halogenated hydrocarbons, and sulfur-containing compounds. Replicate-level semi-quantitative concentrations of all volatile annotations are provided in Table S1, while the mean and standard deviation (SD) of the eight major VOC categories are summarized in Table S2.

**Figure 1:**
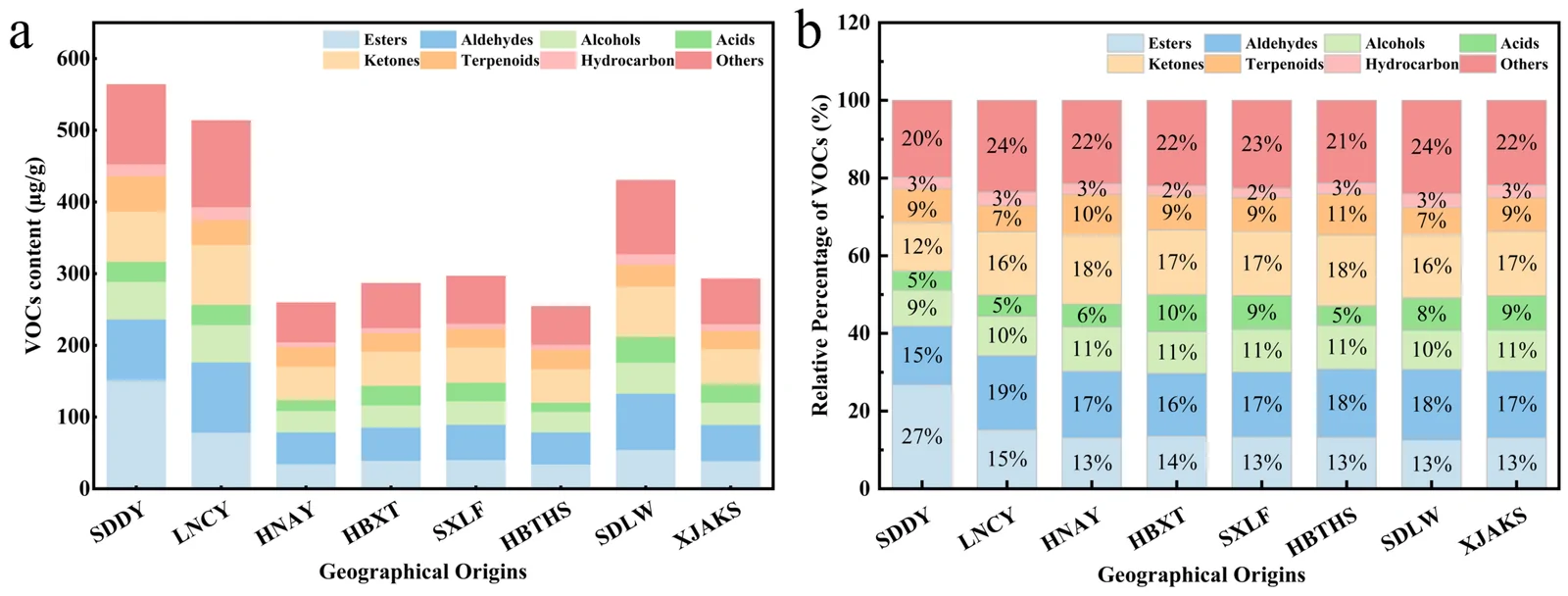
Volatile organic compound (VOC) profiles of commercial sour jujube pulp samples representing eight supplier-reported geographical origins. (**a**) Mean summed semi-quantitative contents (μg/g) of different VOC categories; (**b**) mean relative proportions (%) of the corresponding VOC categories. Six independently prepared analytical replicates were analyzed for each sample group (n = 6). Detailed category-level mean and SD values are provided in Table S2.

Descriptive differences in the summed semi-quantitative VOC contents were observed among the eight sample groups (Figure 1a). SDDY showed the highest mean total VOC content (564.29 ± 24.72 μg/g), followed by LNCY (514.14 ± 18.98 μg/g) and SDLW (430.73 ± 31.37 μg/g). In comparison, lower total contents were observed for HBTHS (254.93 ± 10.02 μg/g) and HNAY (260.13 ± 10.31 μg/g). Because the six measurements within each group represent independently prepared analytical replicates rather than independent orchard-level biological replicates, these differences are described here as variation within the present commercial sample set and are not interpreted as inferential evidence of a causal geographical effect.

The relative distribution of VOC categories also varied among the sample groups (Figure 1b). For each analytical replicate, the relative proportion of a VOC category was calculated from its summed semi-quantitative content divided by the summed content of all VOC categories. Esters showed the most pronounced compositional difference, accounting for 26.78 ± 0.64% of the total VOC content in SDDY, compared with approximately 12.53–15.17% in the other sample groups. Correspondingly, the mean ester content in SDDY reached 151.10 ± 7.00 μg/g, whereas substantially lower ester contents were observed in the remaining groups. Aldehydes, ketones, alcohols, terpenoids, and the “others” category also contributed appreciably to the overall volatile profiles, although their relative distributions differed among sample groups. Detailed category-level values are provided in Table S2.

### 3.2. Principal Component Analysis (PCA) of the Volatile Profiles

**Figure 2:**
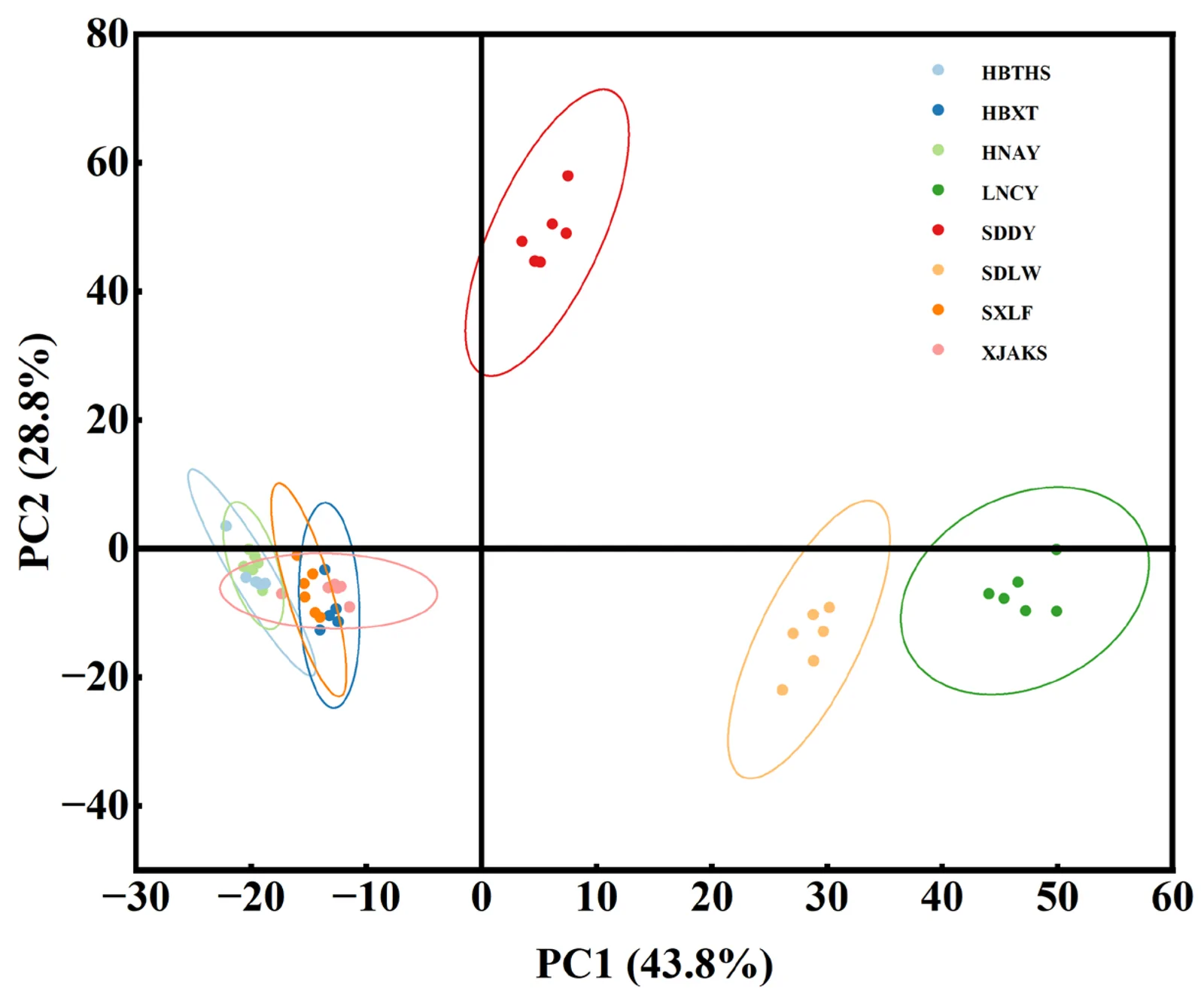
Principal component analysis (PCA) score plot of volatile profiles in commercial sour jujube pulp samples representing eight supplier-reported geographical origins. Each point represents one independently prepared analytical replicate (n = 6 per sample group). PC1 and PC2 accounted for 43.8% and 28.8% of the total variance, respectively.The colors correspond to the eight sample groups indicated in the legend, and the colored ellipses visualize the clustering and distribution of replicates within each group.

To visualize the overall distribution and clustering patterns of the commercial sour jujube pulp samples based on their volatile profiles, an unsupervised principal component analysis (PCA) was performed [25]. As shown in Figure 2, the first two principal components, PC1 and PC2, accounted for 43.8% and 28.8% of the total variance, respectively, corresponding to a cumulative contribution of 72.6%. Thus, the first two principal components captured a substantial proportion of the overall variation in the volatile dataset.

The PCA score plot revealed distinct distribution patterns among several sample groups. SDDY formed a relatively compact cluster in the upper-right region of the score plot and was clearly separated from most of the remaining groups. LNCY and SDLW also exhibited distinct distributions, whereas HBTHS, HBXT, HNAY, SXLF, and XJAKS showed comparatively closer clustering in the lower-left region. These patterns indicate that the volatile profiles differed among the commercial sample groups included in the present study.

Because PCA is an unsupervised exploratory method, the observed separation should not be interpreted as evidence that geographical origin itself caused the differences in volatile composition. In addition, environmental, cultivar, maturity, harvest, and postharvest factors were not independently controlled or statistically partitioned in the present study. Therefore, the PCA results are interpreted as origin-associated differentiation within the present commercial sample set rather than as definitive evidence of geographical causation.

### 3.3. Supervised OPLS-DA and Model Validation

**Figure 3:**
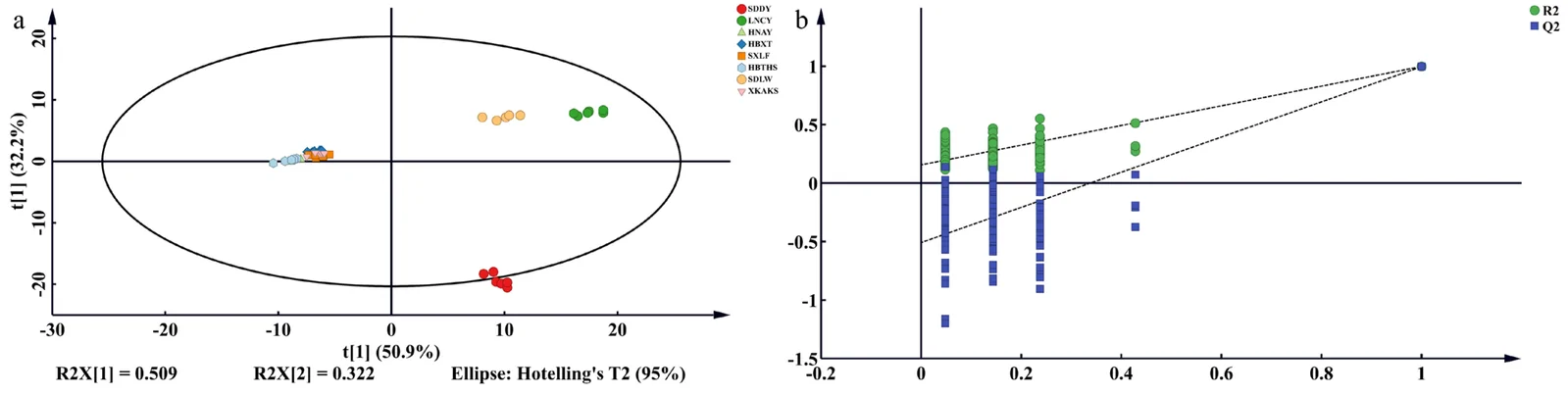
OPLS-DA of volatile profiles in commercial sour jujube pulp samples representing eight supplier-reported geographical origins. (**a**) OPLS-DA score plot based on 48 observations, with six independently prepared analytical replicates per sample group (n = 6). The first and second predictive components accounted for 50.9% and 32.2% of the X-variance, respectively. (**b**) Results of the 200-iteration permutation test. The dashed lines represent the regression lines fitted to the permuted R^2^ and Q^2^ values and extrapolated to the y-axis. The R^2^ and Q^2^ intercepts were 0.156 and −0.508, respectively.

To further evaluate differentiation patterns among the eight supplier-reported geographical groups, supervised orthogonal partial least squares-discriminant analysis (OPLS-DA) was performed using the replicate-level volatile profiles. The model included 48 observations, corresponding to six independently prepared analytical replicates for each sample group. The OPLS-DA model comprised seven predictive components and one orthogonal component, with cumulative R^2^X, R^2^Y, and Q^2^ values of 0.967, 0.891, and 0.811, respectively. In the score plot (Figure 3a), the first and second predictive components accounted for 50.9% and 32.2% of the X-variance, respectively.

The score plot showed clear differentiation among several sample groups. SDDY exhibited a distinct distribution relative to most of the remaining groups, while LNCY and SDLW were also separated from several other groups. In contrast, HBTHS, HBXT, HNAY, SXLF, and XJAKS showed comparatively closer clustering. These results indicate substantial differences in volatile-profile composition within the present commercial sample set. However, because the study did not independently control or partition environmental, cultivar, maturity, harvest, or postharvest factors, the observed separation should be interpreted as group-associated differentiation rather than direct evidence that geographical origin itself caused the differences.

To further assess model stability, a 200-iteration permutation test was performed (Figure 3b). The R^2^ and Q^2^ intercepts were 0.156 and −0.508, respectively. The permuted models generally showed lower model-performance statistics than the original model, and the negative Q^2^ intercept supported the internal validity of the OPLS-DA model. These results indicate no obvious evidence of overfitting under the applied permutation procedure. Nevertheless, no independent external validation set was used; therefore, the model should be regarded as internally validated rather than externally validated.

### 3.4. VIP-Based Discriminatory Volatile Features and Hierarchical Clustering

**Figure 4:**
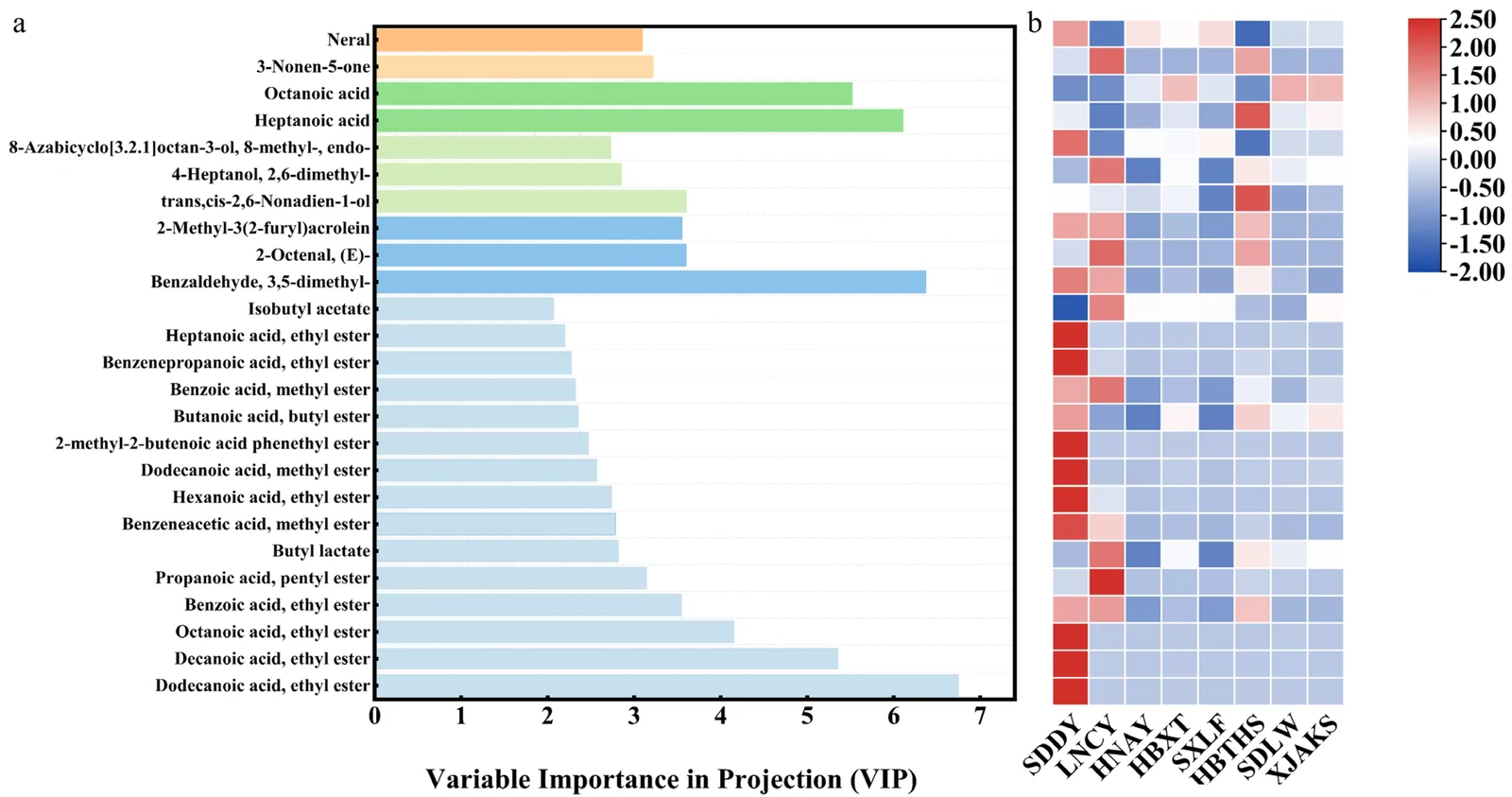
VIP-based discriminatory volatile features and hierarchical clustering patterns in commercial sour jujube pulp samples representing eight supplier-reported geographical origins. (**a**) Variable importance in projection (VIP) scores of selected volatile features derived from the OPLS-DA model; higher VIP values indicate a greater contribution to multivariate group discrimination. (**b**) Hierarchical clustering heatmap showing the relative abundance patterns of the corresponding volatile features across the eight sample groups.

VIP scores derived from the OPLS-DA model were used to rank volatile features according to their contribution to multivariate group discrimination [26]. As shown in Figure 4a, ethyl dodecanoate exhibited the highest VIP score (>6.0), followed by several other high-ranking volatile features, including ethyl decanoate and ethyl octanoate. These VIP-selected features were further visualized using hierarchical clustering to compare their relative abundance patterns among the eight sample groups (Figure 4b).

The heatmap revealed distinct group-associated abundance patterns for several volatile features. In particular, a cluster of medium-chain fatty acid ethyl esters, including ethyl dodecanoate, ethyl decanoate, and ethyl octanoate, showed comparatively high relative abundance in SDDY, whereas lower relative abundances were observed in several other sample groups. This pattern was consistent with the higher ester content observed for SDDY in Section 3.1 and indicates that these ester-related features contributed strongly to the multivariate differentiation of SDDY within the present commercial sample set.

Several aldehydes and alcohols, including (E)-2-octenal and 2,6-dimethyl-4-heptanol, also exhibited group-associated variation in relative abundance. However, these differences should not be interpreted as evidence of up-regulation or activation of specific metabolic pathways, because no transcriptomic, enzymatic, or targeted pathway analyses were performed. Accordingly, the high-VIP features are described here as discriminatory variables or candidate markers rather than validated geographical biomarkers. In addition, VIP-based selection reflects contribution to the multivariate OPLS-DA model and does not by itself establish univariate statistical significance, biological importance, sensory relevance, or causality.

### 3.5. Evaluation of Potential Odor Contribution Based on OAV

Odor activity value (OAV) analysis was further applied to evaluate the potential odor contribution of individual volatile features [20,21] by considering both their semi-quantitative concentrations and reported odor thresholds. Although compound abundance provides important information on volatile composition, compounds occurring at relatively low concentrations may still exhibit high calculated OAVs when their odor thresholds are sufficiently low. However, OAV represents a theoretical estimate of potential odor relevance rather than a direct measurement of sensory perception, because the actual perception of aroma can additionally be affected by food-matrix interactions and interactions among odorants.

After consolidation of duplicate annotations sharing the same quantitative feature, 78 volatile features with OAV ≥ 1 were retained for interpretation, and the complete dataset is provided in Table S3. For concise presentation, representative volatile features exhibiting comparatively high calculated OAVs are summarized in Table 2. A marked distinction was observed between chemical abundance and calculated odor relevance. Medium-chain fatty acid ethyl esters contributed substantially to the overall volatile abundance, particularly in SDDY, whereas several trace volatile features with much lower reported odor thresholds exhibited substantially higher OAVs. In SDDY, 1-nonen-3-one showed a mean calculated OAV of approximately 9.06 × 10^5^. High OAVs were also observed for 2-methoxy-3-(2-methylpropyl)pyrazine and benzenemethanethiol, indicating their potentially important contributions to the aroma profile.

These findings demonstrate that volatile abundance, VIP score, and calculated OAV provide complementary rather than equivalent information for flavor characterization. In particular, high OAVs suggest that low-abundance volatile features may warrant further attention when evaluating the aroma characteristics of sour jujube. Nevertheless, OAV alone cannot establish actual sensory dominance or account fully for synergistic, suppressive, and matrix effects among volatile compounds. Therefore, the sensory relevance of the high-OAV features identified here should be regarded as predictive and requires further validation by sensory evaluation, GC–olfactometry, aroma recombination, and omission experiments [15].

## 4. Discussion

The present study revealed substantial variation in volatile composition among commercial sour jujube pulp samples representing eight supplier-reported geographical origins. By combining multivariate analysis of volatile profiles with odor activity value (OAV) evaluation, the study provided an integrated view of both compositional differentiation and the potential odor contribution of selected volatile features. However, because the samples were commercially sourced and environmental, cultivar, maturity, harvest, and postharvest factors were not independently controlled, the observed differences should be interpreted as origin-associated patterns within the present sample set rather than as direct evidence of geographical or environmental causation.

One of the most prominent compositional features was the comparatively high abundance of medium-chain fatty acid ethyl esters in SDDY, including ethyl dodecanoate, ethyl decanoate, and ethyl octanoate. These ester-related features also ranked highly in the VIP analysis, indicating that they contributed strongly to the multivariate differentiation of SDDY within the present sample set. The accumulation of such esters may be associated with differential activity of alcohol acyltransferase (AAT)-mediated ester biosynthesis during fruit ripening [27]; however, this interpretation remains a mechanistic hypothesis because AAT expression or enzyme activity was not measured in the present study. Accordingly, the observed ester enrichment cannot be directly attributed to specific climatic conditions such as temperature or humidity without targeted environmental correlation analysis.

OAV analysis further indicated that chemical abundance was not necessarily proportional to predicted odor contribution. Several trace volatile features, including 1-nonen-3-one, 2-methoxy-3-(2-methylpropyl)pyrazine, and benzenemethanethiol, exhibited very high calculated OAVs because of their low reported odor thresholds, despite occurring at substantially lower semi-quantitative concentrations than the major ester compounds. This observation highlights the complementary value of OAV analysis in addition to abundance- and VIP-based evaluation. Nevertheless, OAV represents a theoretical estimate based on concentration and odor threshold and does not account for matrix interactions, synergistic or suppressive effects, or human sensory responses [20,21]. Therefore, direct sensory evaluation, GC-O, aroma recombination, and omission experiments would be required to confirm the actual sensory contributions of these volatile features [15].

Several aldehydes and alcohols, including (E)-2-octenal and 2,6-dimethyl-4-heptanol, also showed group-associated differences in relative abundance. Because many C6–C9 aldehydes and alcohols in fruits can arise from lipid-oxidation pathways involving lipoxygenase (LOX)-related metabolism, the observed variation may be consistent with differences in lipid-derived volatile formation [28,29]. However, metabolite abundance alone does not demonstrate up-regulation or activation of the LOX pathway. No transcriptomic, enzymatic, or targeted pathway analyses were conducted in the present study, and therefore the proposed involvement of LOX-related metabolism should be regarded as a hypothesis requiring further validation. Future studies combining volatile profiling with gene-expression analysis, enzyme-activity measurements, and controlled environmental data would be necessary to clarify the biochemical basis of these differences.

## 5. Conclusions

This study characterized the volatile profiles of commercial sour jujube pulp samples representing eight supplier-reported geographical origins using HS-SPME-GC-MS combined with multivariate statistical analysis and odor activity value (OAV) evaluation. Distinct compositional patterns were observed among the sample groups, and several volatile features, particularly medium-chain fatty acid ethyl esters such as ethyl dodecanoate, ethyl decanoate, and ethyl octanoate, contributed strongly to the multivariate differentiation of SDDY samples.

OAV analysis further showed that volatile abundance was not necessarily proportional to predicted odor contribution. Several trace volatile features with low reported odor thresholds, including 1-nonen-3-one, 2-methoxy-3-(2-methylpropyl)pyrazine, and benzenemethanethiol, exhibited high calculated OAVs and may be important aroma-relevant candidates in sour jujube pulp. However, these OAV-based interpretations represent estimates of potential odor contribution and require confirmation by sensory evaluation or GC-O-based approaches.

Overall, the combined use of volatile profiling, multivariate analysis, and OAV evaluation provides a useful approach for characterizing compositional differences among sour jujube samples from different reported origins and for identifying candidate discriminatory and aroma-relevant features. Because the present study was based on commercially sourced samples and did not include independent external validation, orchard-level biological replication, or controlled environmental variables, the findings should be regarded as exploratory evidence of origin-associated differentiation rather than as a validated geographical authentication system. Future studies incorporating independent sample sets, controlled maturity and postharvest conditions, and sensory or molecular validation will be required to assess the broader applicability of these candidate features.

## Figures and Tables

**Table 2:** Representative volatile features with high calculated odor activity values (OAVs) in commercial sour jujube pulp samples representing eight supplier-reported geographical origins.

Compounds	CAS	Odor	Threshold	SDDY	LNCY	HNAY	HBXT	SXLF	HBTHS	SDLW	XJAKS
Benzenemethanethiol	100-53-8	Sharp, alliaceous	3.5 × 10^−6^	32,283.73	23,715.54	30,584.93	27,098.66	2135.96	31,258.44	21,410.96	27,210.69
Non-8-enal	39770-04-2	Smoky, plastic	2.0 × 10^−4^	5417.94	3838.27	5291.49	4592.89	342.86	5364.60	3598.19	4700.48
3-Octen-2-one	1669-44-9	Earthy, spicy	3.0 × 10^−5^	699.29	11,045.65	1332.71	2269.02	206.56	1333.74	4994.39	3300.62
2-Hexenal, (E)-	6728-26-3	Green, grassy	3.1 × 10^−3^	4051.24	3785.54	3764.52	3747.69	85.07	3782.65	3548.94	3987.83
1-Nonen-3-one	24415-26-7	Pungent, mushroom	1.0 × 10^−6^	906,487.70	1658.31	373,082.09	1,093,249.19	72,322.53	303,065.31	1812.00	857,550.23
4-Heptenal, (Z)-	6728-31-0	Oily, fatty	2.5 × 10^−5^	10,191.56	43,663.58	1248.32	2500.24	79.43	1389.74	22,452.48	2236.18
1-Butanol, 3,3-dimethyl-	624-95-3	-	8.2 × 10^−5^	5954.46	14,747.74	1154.25	1453.19	118.03	1155.66	6642.21	1857.37
1-Octen-3-one	4312-99-6	Mushroom	5.0 × 10^−6^	4374.17	31,852.65	1802.83	2429.20	599.54	1528.49	7294.52	3050.39
Butanoic acid, 3-methyl-, 2-phenylethyl ester	140-26-1	Floral, fruity	1.0 × 10^−5^	5915.05	3931.85	5075.56	3776.55	256.21	4594.09	3400.56	4222.12
Pyrazine, 2-methoxy-3-(2-methylpropyl)-	24683-00-9	Green bell pepper, pea	2.0 × 10^−6^	79,581.30	53,402.92	60,855.92	55,452.63	3284.38	60,637.05	43,217.43	58,580.64
Thiazole, 2,5-diethyl-	15729-76-7	Skunk, green	9.0 × 10^−5^	2136.32	2244.37	1876.28	1955.16	136.91	1966.68	1995.42	1861.28
2,4-Decadienal, (E,E)-	25152-84-5	Dusty, waxy	7.0 × 10^−5^	1105.05	3355.21	1112.81	1282.23	86.98	853.79	2043.12	3147.36
Damascone,.beta.-	23726-91-2	Fruity, floral	2.0 × 10^−6^	17,262.42	30,803.69	2423.88	2740.46	440.75	2827.83	17,139.11	2462.19
Furaneol	3658-77-3	Sweet, cotton	1.0 × 10^−3^	4241.19	3763.04	4212.73	4376.23	339.86	4416.19	4054.48	4251.11
Ethyl 3-cyclohexenecarboxylate	15111-56-5	-	3.0 × 10^−6^	5804.59	18,512.98	2579.65	8313.81	466.93	2639.37	19,747.49	6803.41
2,6-Nonadienal, (E,Z)-	557-48-2	Cucumber, green	1.0 × 10^−5^	7458.27	33,429.72	3211.22	5895.15	488.10	4067.49	30,548.86	3962.05
2-Butanethiol, 3-methyl-	2084-18-6	Repulsive, beefy	2.0 × 10^−4^	1038.83	2827.38	505.12	641.72	86.18	469.49	989.51	537.53
trans,cis-2,6-Nonadien-1-ol	28069-72-9	Green, cucumber	1.0 × 10^−3^	1547.81	1439.14	1381.20	1246.34	228.40	984.38	2151.87	1128.75
Phenol, 3-ethyl-	620-17-7	musty	8.5 × 10^−4^	9422.09	12,996.26	2210.39	3841.79	513.78	1820.21	13,398.58	3664.28
Cyclohexanone, 2,2,6-trimethyl-	2408-37-9	Pungent, thujone	1.0 × 10^−4^	622.95	861.27	986.05	1402.87	155.86	1172.74	948.96	1209.97

Notes: Values are mean calculated OAVs based on six independently prepared analytical replicates (n = 6) per sample group. OAVs were calculated using semi-quantitative concentrations. The compounds listed here were selected as representative high-OAV volatile features for concise presentation; the complete dataset of features with OAV ≥ 1 is provided in Table S3. OAV indicates potential odor contribution and does not constitute a direct sensory measurement.

## Data Availability

The original contributions presented in this study are included in the article/Supplementary Materials. Further inquiries can be directed to the corresponding authors.

## Supplementary Materials

The following supporting information can be downloaded at: https://www.mdpi.com/article/10.3390/foods15183318/s1, Table S1. Replicate-level semi-quantitative concentrations of volatile features detected in commercial sour jujube pulp samples representing eight supplier-reported geographical origins. Table S2. Mean and standard deviation (SD) of absolute contents and relative proportions of major volatile organic compound (VOC) categories in commercial sour jujube pulp samples representing eight supplier-reported geographical origins. Table S3. Calculated odor activity values (OAVs) of volatile features with OAV ≥ 1 in commercial sour jujube pulp samples representing eight supplier-reported geographical origins. Table S4. Identification information for volatile features detected in commercial sour jujube pulp samples by HS-SPME-GC-MS. Table S5. Retention-time and experimental retention-index information for 30 representative volatile features in commercial sour jujube pulp samples. Table S6. Peak areas of volatile features detected in the system blank vial.

## Abbreviations

The following abbreviations are used in this manuscript:

AAT	Alcohol Acyltransferase
CAS	Chemical Abstracts Service
GC-MS	Gas Chromatography-Mass Spectrometry
HS-SPME	Headspace Solid-Phase Microextraction
LOX	Lipoxygenase
OAV	Odor Activity Value
OPLS-DA	Orthogonal Partial Least Squares-Discriminant Analysis
PCA	Principal Component Analysis
VIP	Variable Importance in Projection
